# Timber harvesting intensity and use of decision-support tools among semi-professional private forest owners: a Norwegian case study

**DOI:** 10.1038/s41598-026-55795-0

**Published:** 2026-06-15

**Authors:** Hanne K. Sjølie, Ane Christensen Tange

**Affiliations:** 1 Department of Forestry and Wildlife Management, Faculty of Applied Ecology, Agricultural Sciences and Biotechnology, University of Inland Norway (INN), Evenstad, Norway; 2 Glommen Mjøsen Skog SA, Pb 1329, Elverum, 2405 Norway

**Keywords:** Harvest predictions, Forest management plan, Management objectives, Timber resource, Forest inventory, Non-industrial private forest owners, Ecology, Ecology, Environmental sciences

## Abstract

Non-industrial private forest owners are known to have multiple ownership values and objectives. Their management decisions have multiple impacts on the supply of ecosystem services from forests. Forest management plans, a main decision-support tool for many forest owners, tend to be timber-oriented, potentially leading to more harvesting and more frequent use among production-oriented owners. We investigated factors explaining harvest intensity, measured as the ratio of property-level actual harvest volumes to predicted harvest volumes and the use of forest management plans among 119 semi-professional, non-industrial, private forest owners in Norway. Harvest intensity decreased with forest area and with biodiversity and nextgeneration’s need as ownership objectives and increased with wood prices and owner engagement but was not impacted by short-term profit or use of forest management plans. Use of forest management plans increased with forest area, having received instructions, trust in the harvest predictions, and economic ownership objectives. By using property-level harvest prediction, we could compare harvest figures to the actual timber resource, formed by each property’s forest biophysical attributes. This approach may be a valuable step in developing models that provide enhanced understanding of how forest owner behavior is shaped by preferences and objectives.

**Supplementary Information:**

The online version contains supplementary material available at https://doi.org/10.1038/s41598-026-55795-0.

## Introduction

The boreal forests constitute the world’s largest terrestrial biome, encompassing 27% of the world’s forests^1^. The species diversity of boreal forests is generally lower than of other forest ecosystems, but they still include an important part of the world’s biodiversity; not at least due to the intactness^2^. These forests supply goods and services pivotal to human wellbeing^3,4^ including a substantial part of the global timber harvest^5^. Boreal forest countries are also placed among the world’s main producers of sawnwood and paper^6^.

In many European countries and in the United States, non-industrial private forest owners (NIPF) make up a substantial share of forest ownership^7^. Generally, these owners are known to be not mere profit-maximizers, but have multiple objectives related to timber and non-timber goods^8^; the values of amenities can be substantial^9^. NIPF owners can be divided into groups depending on how they value various goods and services^10^. The literature on forest owners’ attitudes toward their forest and its management is rich, with the vast majority of analyses being stated-preferences studies^11^. Revealed-preferences studies show that forest owners’ economic vs. non-economic objectives impact the decision to harvest and the harvest volumes^10,12,13^. However, the lion’s share of revealed-preferences studies only partially account for the forest resource base, typically by including a variable for forest land area. Thompson et al. (2017)^14^ and Butler and Sass (2023)^15^ are notable exceptions. In a study of the northern United States, Thompson et al. (2017)^14^ modeled harvest intensity defined as share of the live basal area removed within a period on a national forest inventory plot. They found that harvesting intensity depends on a combination of biophysical and social factors, like owner type, live basal area, household income and distance to road. Butler and Sass (2023)^15^ also combined social and biophysical data to derive factors explaining whether harvest took place on a national forest inventory plot. They found that previous income from the forestland, receiving management advice, region, basal area and softwood area impact the probability of harvest taking place. These studies represent important steps forward in including the biophysical resource in greater detail into behavioral models. Yet, for the forest owner, the productive forest land base as a whole forms the decision basis as owners can spatially trade off harvest across stands and parcels.

A forest management plan (FMP) provides managers with information to support strategic, tactical and operational forest management planning^16^. Due to its importance for forest management, FMP also functions as a forest policy instrument to support management and harvest in many countries^17^. Having a FMP has been shown to increase the probability that an owner harvests^13^. Also, during periods with high timber prices and competition for timber among buyers, FMP can provide forest owners with an important support to avoid hasty harvesting with negative impacts on the future timber resource, biodiversity and aesthetics^18^.

Despite their importance, there is large variation in the share of owners who have an FMP. In the United States, 11% of family forest owners, collectively owning 24% of forest land, have a written FMP^19^. In Sweden, FMPs exist for 66% of the productive forest area owned by individual forest owners^20^; owners may choose management recommendations based on standardized timber production or with more consideration for environmental attributes^21^. In Finland, 63% of the privately owned forest area is covered by FMP^22^. In Norway, by contrast, about 37% of the NIPF have an FMP; the probability of having an FMP increases with forest land area^16^. However, only 40% of Norwegian forest owners with an FMP implement its proposals; implementation rate increases if the FMP is adapted to forest owners’ objectives^16^. In addition, FMPs older than five years have been found to be used less frequently^18^.

There are important gaps in the literature regarding how non-timber values impact forest owners’ management decisions. Most studies that have investigated factors steering actual harvest behavior do not account directly for the harvest potential of the owner’s forestland. While “potential” can be understood and defined in multiple ways, the timber resource available for timber supply is arguably steering how much the owner can harvest. The timber resource base can be understood as a function of growing stock, growth of harvestable timber, accessibility based on terrain attributes and availability determined by regulations. Thompson et al. (2017)^14^ modeled harvest intensity defined as share of the live basal area removed within a period on a national forest inventory plot. However, owners typically have multiple management units or stands and do see these together in management decisions. To the best of our knowledge, no studies have used forecasted or predicted harvest levels based on inventory data to benchmark actual harvest levels. By comparing actual timber harvest to predictions, harvest volumes are contrasted to the growing stock, taking future growth, harvest maturity, access and availability into account. Such analyses can provide more accurate estimates of forest owner behavior and new insight into how forest owners manage their forestland.

FMP is a major policy instrument and often the main management decision-support tool that owners have at hand. Despite its importance, very few studies have investigated the actual use of FMP. One exception is Bashir et al. (2024)^16^, who found that in Norway, a minority of the forest owners that have a plan uses it and follow its advice. However, FMP management recommendations tend to be designed to support timber-production policies^16^. With the knowledge that many forest owners are multi-objective, this raises the question whether the use of FMPs is biased toward owners with timber production and economic ownership objectives.

We attempt to fill parts of these voids by constructing regression models to (1) test whether the known relationships between ownership objectives and harvest levels hold when considering predicted harvest levels, and (2) explain the use of FMP for steering harvest decisions. Based on the literature, we draw the following hypotheses:

H1: Including the ratio of actual-to-predicted harvest levels validate the established relationships between ownership objectives and harvest levels

H2: The use of FMP increases harvest intensity

H3: Forest owners with economic ownership objectives use FMP more than others

For testing these hypotheses, we use a unique triangulated dataset consisting of property-level harvest predictions, property-level actual harvested volumes, and a survey of forest owner objectives and FMP use.

## Methods and materials

### Forest management plans in Norway

In Norway, FMPs are not mandatory, not even for selling timber. However, subsidies to new FMP are a key forest policy instrument to support “sustainable forestry with active commercial exploitation of forest and outfield resources in the short and long term”^23^ (§ 1). Detailed requirements for data sampling scope and methods, functions and data formats in FMP are set by the public authorities^23^. Direct subsidies may cover up to 50% of FMP costs^24^. In addition, forest owners receive tax deductions on the FMP purchase^23^. Thus, forest owners’ share of total costs may vary from 4 to 30% depending on the owner’s marginal income tax rate^25^. Industrial timber buyers only purchase timber from properties that have undergone mapping of a set of environmental attributes for set-aside, and this mapping usually takes place together with the timber resource inventory as part of the FMP data collection. However, despite considerable incentives, participation rates vary from about 50% to 90% of the area depending on i.a. local forestry traditions (Geir Korsvold, personal communication, March 16, 2021). With the prevalence of small forest owners in Norway^26^, new FMPs are typically made for a municipality or part of a municipality where all forest properties above a given minimum size are invited to join. The county governor initiates new FMP for an area^27^ with typical FMP frequency of 15 years. The FMP projects are led by a committee that consists of the municipality administration’s head of forestry, together with representatives from timber buyer organizations, forest owners in the area and the county governor. The committee decides on the conditions in the competitive tendering, where FMP suppliers are invited to submit an offer, within the frames of the regulations. The FMP design varies with available data, economic support and the local FMP committee’s requirements. In essence, it typically consists of stand-level inventories that are based on remotely sensed data, usually airborne-laser scanning^28^. To complement the remotely sensed data, field data may be gathered. Inaccessible and set-aside areas are subtracted from inventories and predictions of property-level annual harvest quantity for the next 3–4 decades may be produced. The harvest prediction is based on the inventory data that are fed into a management and harvest simulator and usually maximizes net present value with the constraints of maximum sustainable timber yield, i.e., harvest levels should be non-declining^29^. In our study, FMPs consist of property-level inventory registers together with timber harvest predictions.

### Study area

The county of Innlandet in Norway was chosen as the study area because it supplies almost 40% of the domestic harvest for commercial sales and contains 26% of the country’s productive forest area^30^. Further, the county has a variation in forestry practice from small scale forestry in mountainous areas to intensive forestry in the lowlands. Forests consist of the boreal conifer species Norwegian spruce (*Picea abies*) and Scots pine (*Pinus sylvestris*) with a mix of boreal broadleaves, such as birch, (*Betula *spp.) alder (*Alnus* spp)., willow (*Salix* spp). and aspen (*Populus tremula*). There are in total 20 545 forest properties in Innlandet owning together 1.8 mill. hectares productive forestland^26^. Almost 69% of the productive forest area in Innlandet is owned by NIPF owners, with a mean forest property size of 60 hectares^31^.

**Fig. 1:**
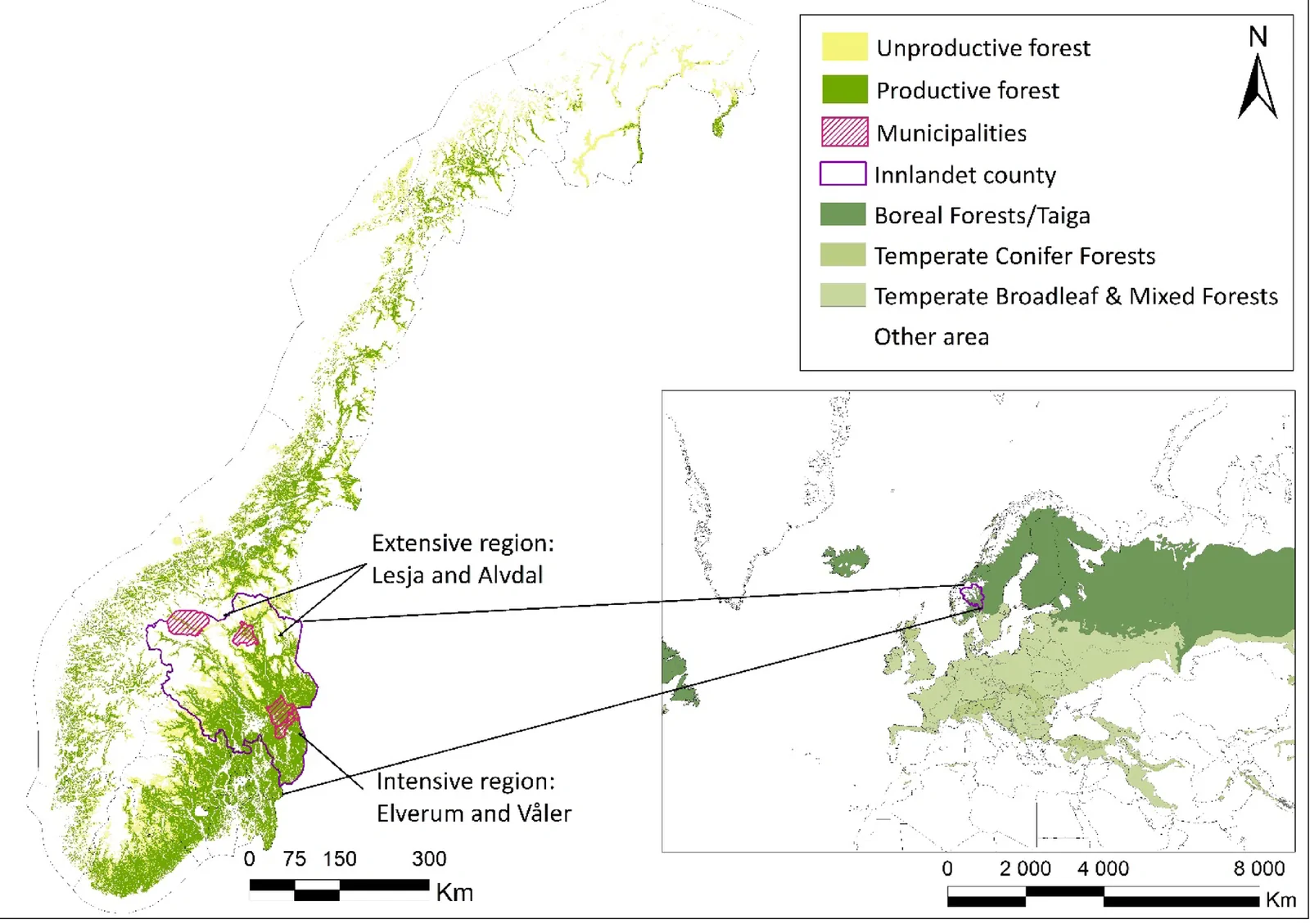
Map of Norway showing the sampled municipalities. The green and yellow colors display the extent of forest in Norway^33^. The smaller map shows the study areas placement in Europe, located in the boreal forest (dark green)^34^. The map was created with the software ArcGis Desktop version 10.8.2, available at https://www.esri.com/en-us/arcgis/.

To compare actual harvest levels with predicted harvest levels, we selected forest owners who had acquired FMPs from the years 2017–2019 with predicted harvest levels. To include owners from areas with intensive and extensive forestry, four municipalities in Innlandet county were included (Fig. 1; Table 1). The main management practice is even-aged rotation forestry in both regions. The intensive region is characterized by active forest management, with soil scarification followed by planting, seeding or natural regeneration and precommercial thinning and short rotations. The extensive region is characterized by mountain forestry management with smaller clearcuts and longer rotation periods largely relying on natural regeneration following harvesting. There are several timber buyer companies present in the intensive region, while only one-two buyers are present in the extensive region.

**Table 1:** Overview of productive forest area by municipality and Innlandet county^26^, year of forest management plan (FMP), and total harvested timber volume (1000 m^3^ yr^-1^), harves volumet per hectare (m^3^ ha^-1^ yr^-1^) and gross value (mill. € yr^-1^). All numbers are averages for the period 2018-2024^32^.

Management region	Municipality	Year of FMP	Productive forest area (1000 ha)	Harvested timber for sale		
				Total harvest volume (1000 m^3^ yr^− 1^)	Harvest volume per hectare (m^3^ ha^− 1^ yr^− 1^)	Gross value (mill. € yr^− 1^)
Intensive	Elverum	2017	91.5	341.2	3.73	14.9
Intensive	Våler	2017	50.7	138.2	2.73	5.9
Extensive	Lesja	2019	15.1	13.4	0.89	0.5
Extensive	Alvdal	2017	27.0	32.8	1.21	1.1
-	Innlandet county	-	1891.3	4547.6	2.40	173.6

### Data

A questionnaire was developed in the online service Nettskjema.no, designed for survey data gathering in Norwegian research. The survey was sent to all NIPF owners who had bought FMPs with harvest predictions from Glommen Mjøsen Skog in the years 2017 and 2019 in the four municipalities listed in Table 1, totaling 591 individuals, excluding properties under common ownerships that were not spouses. Survey requests were sent by e-mail with a link to Nettskjema, accessible from March 21 to August 1, 2024. Within this period, two follow-up emails and one SMS were sent to owners who had not responded. After removing owners that had sold the forest, deceased owners, invalid e-mail addresses and properties that lacked predictions, the survey gross sample consisted of 575 owners and their properties.

The survey collected information on forest owners’ harvest decisions, the use of forest management plans and management goals (Table 2). The respondents were asked to rank their agreement with given statements on 4-point Likert scales^35^. The questionnaire also gathered consent to collect harvest data from public registers and harvest prediction data from FMP available from Glommen Mjøsen Skog’s databases. The full questionnaire is included in Supplementary 1 (original in Norwegian) and Supplementary 2 (translated to English).

As data collection was restricted to four municipalities with new FMPs (Table 1), a forest property was defined as all forest within one municipality with the same owner(s). For each forest property, harvest predictions were taken from the FMP. FMPs provide data on the property’s forest resources and environmental values and property-level yearly predicted harvest levels. The FMPs used in this study were delivered to owners by Glommen Mjøsen Skog which had the responsibility for field data collection, harvest level simulation and delivery of the final FMP product to the forest owners.

The harvest predictions were simulated on the basis of remote sensing and field forest data fed into a simulation-optimization model framework based on the Avvirk model^29^. Stand data were run in a scenario-based simulator which uses the recorded forest attributes as input (e.g. age class, site index, stand age, number of trees, volume of spruce, pine and broadleaves, height, productive area and forest type) together with simulated forest growth and mortality dynamics. Scenarios are simulated for possible combinations of management intensity, thinning and final harvest, typically for five periods of ten years each. The optimization model finds the scenario that maximizes the net present value based on preset criteria and parameters like interest rate, timber prices and operational costs as well as non-declining timber harvest. The final result to the forest owner is harvestable timber volume for the property.

Data on harvest volumes and wood prices were collected from the County Governor of Innlandet’s registers of yearly property-level harvest for commercial sales in the period 2018–2024, i.e., seven years with the exception of Lesja municipality, where harvest data ranged from 2020 to 2024, i.e., five years. Harvest data were summarized as yearly total volume in cubic meters by forest property. Wood prices were weighted by assortment volume, owner and year. The municipality means of wood prices were assigned to owners with no harvest.

Outliers were visually detected^36^ and two observations that differed considerably from the others in terms of harvest intensity over productive forest area, were removed.

### Ethical considerations and participant consent

The collection of survey and harvest data, along with the use of FMP data, and the alignment of these three datasets were approved by the Norwegian Agency for Shared Services in Education and Research (SIKT) on January 31, 2024, and received retrospective institutional ethical approval from the University of Inland Norway on October 15, 2025. The three datasets were linked using an ID key, which anonymized the complete dataset for further analysis, ensuring that all personal information was excluded. This process complies with national data protection regulations and follows the University of Inland Norway’s guidelines for research data. Informed consent was obtained from all forest owners who participated in the survey. Participants received detailed information about the purpose of the data collection, how the data would be handled, and were asked to provide their consent (Supplementary 2). Access to the forest owner data is strictly limited to the authors and is not shared with external parties, including other people at Glommen Mjøsen Skog SA.

**Table 2:** Description of data used in the two models: Harvest intensity and use of FMP.

Category of variables	Variables	Source	Description of variables	Type of variable
Dependent variable	Harvest intensity	FMP, Harvest registers	∑ actual harvest / ∑ predicted harvest volume	Numeric
	Use of FMP	Survey	Use of proposed harvest volumes to decide the harvest level over several years	Ordinal (1–4 points)
Owner data	Owner age	Survey		Numeric
	Gender	Survey		Categorical
	Engagement	Survey	Composed of the four statements “I watch the state of the forest” + “I observe timber prices” + “I study technical questions about forestry” + “I care about proper management of the forest”	Ordinal (4–16 points)
Economic factors	Wood price	Harvest register	Wood price	Numeric
	Forest area	FMP	Productive forest (hectares) in logarithmic scale	Numeric
Ownership objectives	Biodiversity objective	Survey	Biodiversity	Ordinal (4-point Likert scale): Of very little importance Of little importance Of considerable importance Of decisive importance
	Nature goods objective	Survey	Nature goods	
	Next generation objective	Survey	Next generation’s needs	
	Recreation Hunting	Survey	Recreation and hunting	
	Short-term profit objective	Survey	Short-term harvest and economic profit	
	Long-term profit objective	Survey	Long-term harvest and economic profit	
	Economic security objective	Survey	Economic security	
	Stable harvest level objective	Survey	Stable harvest level	
	Economic objective	Survey	Short-term profit + Long-term harvest profit + Economic security + Stable harvest level	Ordinal (4–16 points)
Information	FMP Instruction	Survey	Did you participate in any forest management activities upon purchasing the forestry plan?	Binary: Yes No
Assessment	Assessment of predictions	Survey	Yes if agree that the predictions yield about a right harvest level (between 10% too high and 10% too low), otherwise no	Binary: Yes No

### Statistical analyses

We created two regression models. The first model was created to explain Harvest intensity, defined as

$$\:{Harvest\:Intensity}_{i}=\:\frac{\sum\:_{t}{\mathrm{y}}_{\mathrm{i},\mathrm{t}\:}\:}{\sum\:_{t}{\mathrm{k}}_{\mathrm{i},\mathrm{t}\:}\:}$$

Where

y_i, t_ = actual harvested timber volume on property i in year t

k_i, t_ = predicted timber harvest volume on property i in year t

Since most owners do not harvest annually, actual and predicted harvest volumes were summed over the period since the FMP were shared with the owners (5 years for Lesja municipality; 7 years for the other municipalities).

Due to the zero-censoring of harvest intensity, a Tobit model was constructed. Harvest intensity served as the dependent variable in the Tobit regression model^37^ with independent variables listed in Table 2.

Let the latent harvest intensity be:

1 $$\mathrm{H}_{i}* = ~\beta _{0} + ~X_{i} \beta + \mu _{i} ,$$

and the observed harvest intensity be

2 $$\mathrm{H}_{i}= \{\mathrm{H}_{i}*{\text{ if}}\,\,\mathrm{H}_{i}*>0; \,0\,\text{ if}\, \mathrm{H}_{i}*\leq0\}$$

Where the vector X_i_ contains the independent variables, $\:{\upbeta\:}$ the coefficients to be estimated and $\:{{\upmu\:}}_{\mathrm{i}}$ the error terms.

The second model seeks to unveil the use of FMP to determine harvest levels over multiple years. Including all usage levels in the four-point Likert scale, we created an ordered (cumulative) logit regression model. The model is based on the assumption that the use of FMP can be expressed as

3 $$y*_{i} = X_{i} \beta + \mu _{i} ~$$

Where y*_i_ is continuous and unobservable use of FMP, X_i_ is the vector of indepedent variables, $\:{\upbeta\:}$ the coefficients and $\:{\mu}_{i}$ the error term. Even if the use of FMP for determining harvest level is not observable, we do observe Likert scale levels, Y^38^. Thus, assuming that ρ_j_ constitute the cut-off points of $\:\mathrm{y}_i\mathrm{*}$, we have the following relationship between the latent variables and the observations Y:

4 $$\begin{gathered} Y_i = {\text{ }}1{\text{ }}{\text{ if}}\,{\text{ }}y_i* \le \rho _{1} \hfill \\ Y_i = {\text{ }}2{\text{ }}{\text{ if}}\,\rho _{1} < y_i* \le \rho _{2} \hfill \\ Y_i = {\text{ }}3{\text{ }}{\text{ if}}\,\rho _{2} \hfill \\ Y_i = {\text{ }}4{\text{ }}{\text{ if}}\,\rho _{3} >y_i* \hfill \\ \end{gathered}$$

With this model, the cumulative probabilities of having an observation in any given level are estimated. The impact of the individual variables on the use of FMP was assessed through cumulative (log-odds ratios) (logits)^39^:

5 $$Logit[(P\left( {Y_i{\text{ }} \le {\text{ }}j} \right)]{\text{ }} = \log \bigg[\frac{{P\left( {Y_i \le j} \right)}}{{P(Y_i > j)}}\bigg],\,\quad j{\text{ }} = {\text{ }}1,\,2,\,3$$

Maximum likelihood estimation was employed for estimating the coefficients, by the polr function in R (2025.05.1), MASS package^40^. $\:\beta\:$ is assumed constant across levels (parallel slopes)^37^; this assumption (proportional odds assumption) was tested using the Brant test. The lowest p-value was 0.295, thus the assumption of proportional odds was not violated.

Several models with the available variables that were considered the most relevant from literature review and theory were tested. Collinearity was tested with parametric (Pearson) and non-parametric tests (Spearman correlation), alpha = 0.05, as well as VIF values (VIF > 5) in R package ‘car’ and visual inspection. Nagelkerke’s pseudo R^2^^41^ was used to test model fitness and determine the final choice for the two regressions models.

## Results

### Descriptive statistics

**Fig. 2:**
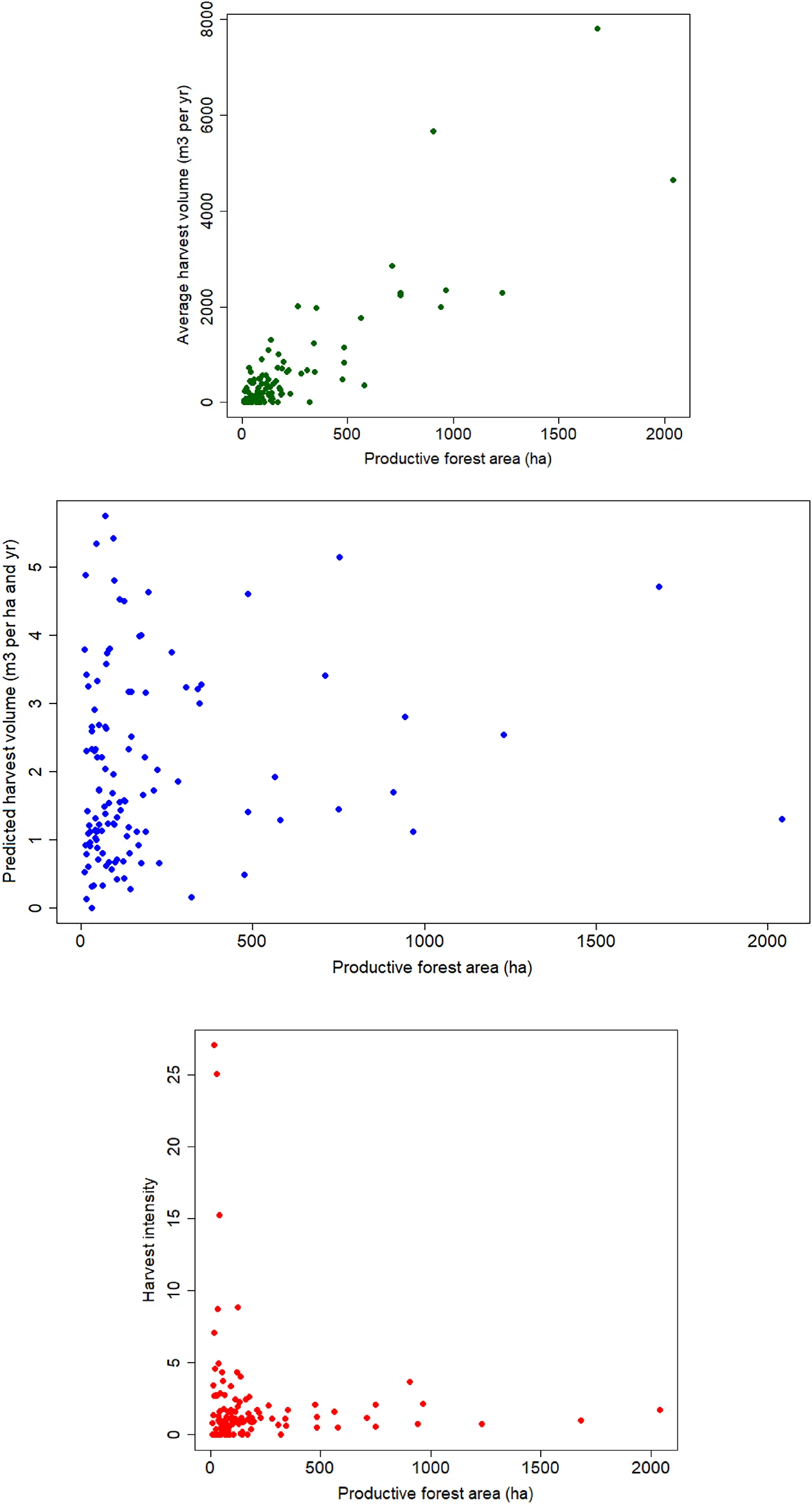
Top: Average harvest volume (m^3^ yr^-1^ over productive forest area (ha). Middle: Predicted volume (m^3^ ha^-1^ yr^-1^ over productive forest area (ha). Bottom: Harvest intensity (Sum of harvest volume divided by the sum of predicted volume) over productive forest area (ha).

The final dataset consisted of 119 respondents, given a response rate of 22%. All questions used in the analyses were answered by all respondents. The mean productive forest area among all respondents was 193.5 ha, ranging from a minimum of 95.9 ha to a maximum of 2041.2 ha with a right-skewed distribution (Table 3; Fig. 2). While all properties had a prediction volume above 0, ranging from 2 m^3^ to 7914 m^3^ yr^− 1^, 19.3% of owners did not have any harvest for sale in the prediction period. Owners were between 32 and 84 yearsat the time of the survey with mean owner age being 60 years. Twenty percent of the respondents were female. A total of 46.2% of owners had forestry or agricultural education, either at the secondary or tertiary level or from vocational training. Of all owners, 42.0% (50 owners) had their forest located in the extensive region. In addition, 58% of respondents stated that they had received instruction during release of the FMP. Out of the eight ownership objectives, long-term profits and next generation’s needs were the most important. Short-term profits and recreation and hunting were considered least important, with no owners stating short-term profits to be of decisive importance. Economic security, stable harvest levels, biodiversity and nature goods all fall in the medium-importance range.

**Table 3:** Descriptive statistics. *N* = 119 for all variables.

	Min.	Median	Mean	Max.	SD
Actual harvest volume in the period (m^3^ yr^− 1^)	0	184.6	554.1	7804	1087.8
Predicted harvest volume in the period (m^3^ yr^− 1^)	2	139.0	448.7	7914	950.8
Harvest intensity	0	1.0	1.9	27.1	3.8
Productive forest area (ha)	9.59	91.6	193.5	2041.2	310.8
Ln (Productive forest area)	2.26	4.52	4.56	7.62	1.13
Owner age	32	60	60.2	84	12.2
Engagement	7	13	13.0	16	2.42
Wood price (NOK/m3)	253.4	474.2	482.6	686.8	140.6
Ownership objective: Biodiversity	1	2	2.4	4	0.87
Ownership objective: Nature goods	1	2	2.4	4	0.84
Ownership objective: Next generation	1	3	2.7	4	0.88
Ownership objective: Recreation hunting	1	2	2.0	4	0.84
Ownership objective: Short-term profit	1	2	2.0	3	0.77
Ownership objective: Long-term profit	1	3	2.6	4	0.86
Ownership objective: Economic security	1	3	2.4	4	0.87
Ownership objective: Stable harvest level	1	2	2.3	4	0.88
Ownership objective: Economic	4	10	9.3	15	2.36

Average yearly harvest volume was correlated with productive forest area (Fig. 2 top). Predicted harvest volume per area unit was only weakly correlated with productive forest area (Fig. 2 middle), correlation coefficient was − 0.16. Plotting the harvest intensity against productive forest area displays a negative, exponential relationship (Fig. 2 bottom). Subsequently, the maximum harvest-prediction ratio was 27.1. Minimum ratio value was 0, observed among 19.5% of the respondents.

### Harvest intensity

Factors explaining harvest intensity, measured as the ratio of total actual harvest to total predicted harvest volume for the same period, are given in Table 4. A larger productive forest area led to lower harvest intensity, while owner engagement had the opposite impact. A decrease in owner age of ten years or an increase in timber price of 50 NOK (~ 10 €) would each imply a higher harvest intensity of about 0.5. One point change on the Likert scale of biodiversity importance suggests a decrease of harvest intensity of 0.5 while the same increase in next generation needs reduces harvest intensity by 0.33. The variables use of FMP, short-term profit as management objective and owner gender did not impact harvest intensity. The model’s pseudo R^2^ was 0.13.

**Table 4:** Tobit regression output of harvest intensity. Harvest intensity = Actual harvest volume divided by predicted volumes in predictions over the analyzed period. Significance levels (* = 0.1, ** =0.05 or *** = 0.01) refer to significant impact of the response variables on harvest intensity.

	Estimate	SE	z value	p value
(Intercept):1	2.665	1.993	1.337	0.181
(Intercept):2	0.750	0.083	9.057	< 2e-16***
Owner age	− 0.048	0.019	− 2.583	0.010**
Gender (M)	− 0.390	0.537	− 0.726	0.468
Forest area (log)	− 0.786	0.219	− 3.587	< 0.001***
Engagement	0.213	0.098	2.180	0.029*
Wood price	0.010	0.002	5.260	< 0.001***
Biodiversity objective	− 0.510	0.259	− 1.973	0.048*
Next generation objective	− 0.327	0.263	− 1.242	0.214*
Short term profit objective	− 0.034	0.283	− 0.120	0.904
Use of FMP	0.167	0.303	0.552	0.581

### Assessment and use of forest management plan

**Fig. 3:**
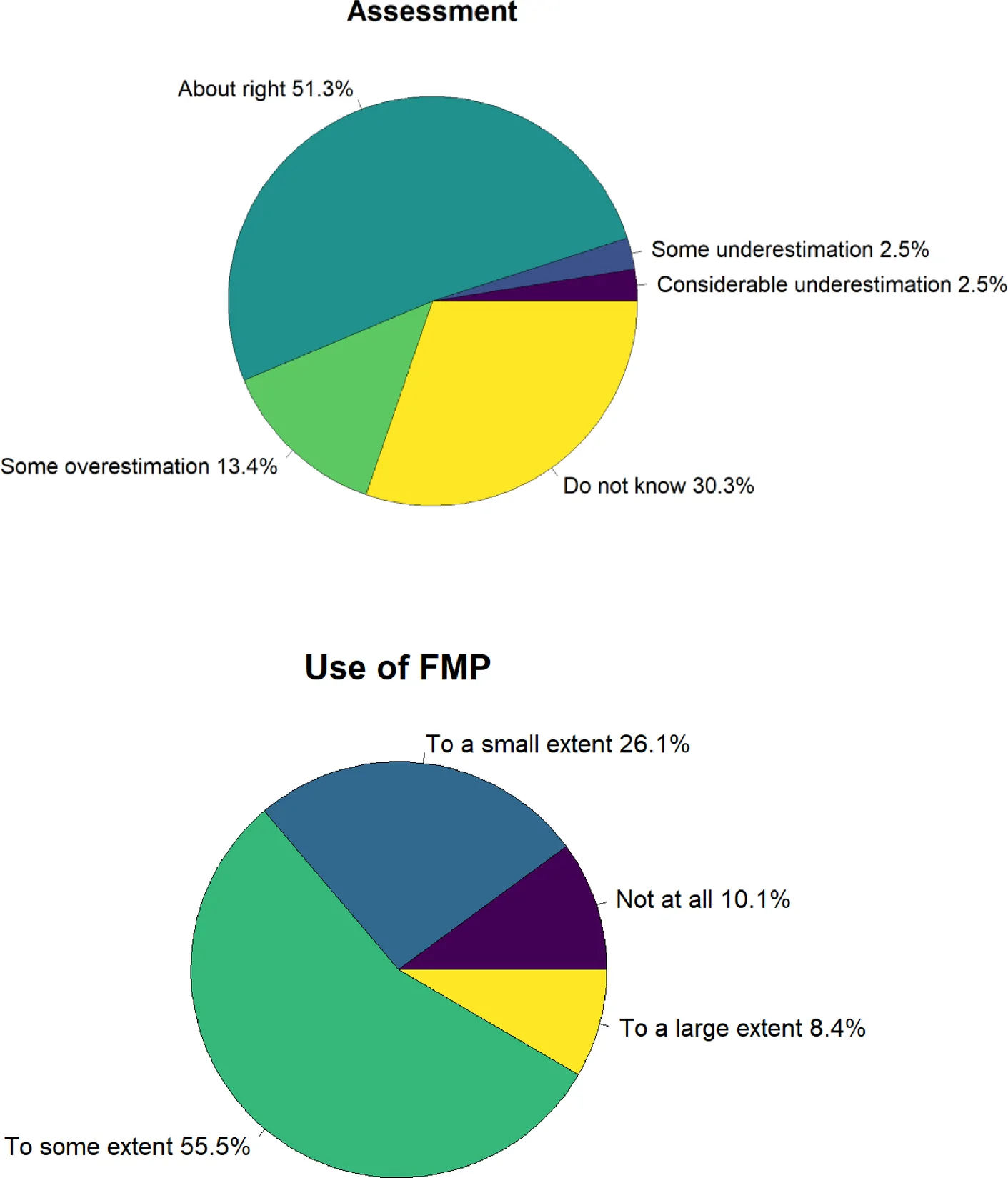
Top: Assessment of harvest predictions’ accuracy (percent of respondents). Bottom: Use of FMP’s harvest predictions (percent of respondents).

51% of the respondents judged the volume predictions to be about correct and yielding predictions levels between 10% too low and 10% too high. Out of those that said that the predictions fell outside the 10% margins, 73% said the volumes were overestimated. However, 30% of the respondents stated that they do not know if the predictions’ timber yields reflect a reasonable harvest level (Fig. 3 top). Out of the 119 respondents, 10% stated that they do not use the predictions to steer timber harvest volume at all. Further, 26% said that they use the predictions to a limited degree, while 55% used them to some degree. In total, 8% said that they to a large degree base timber harvest volumes on the predictions (Fig. 3 bottom).

**Fig. 4:**
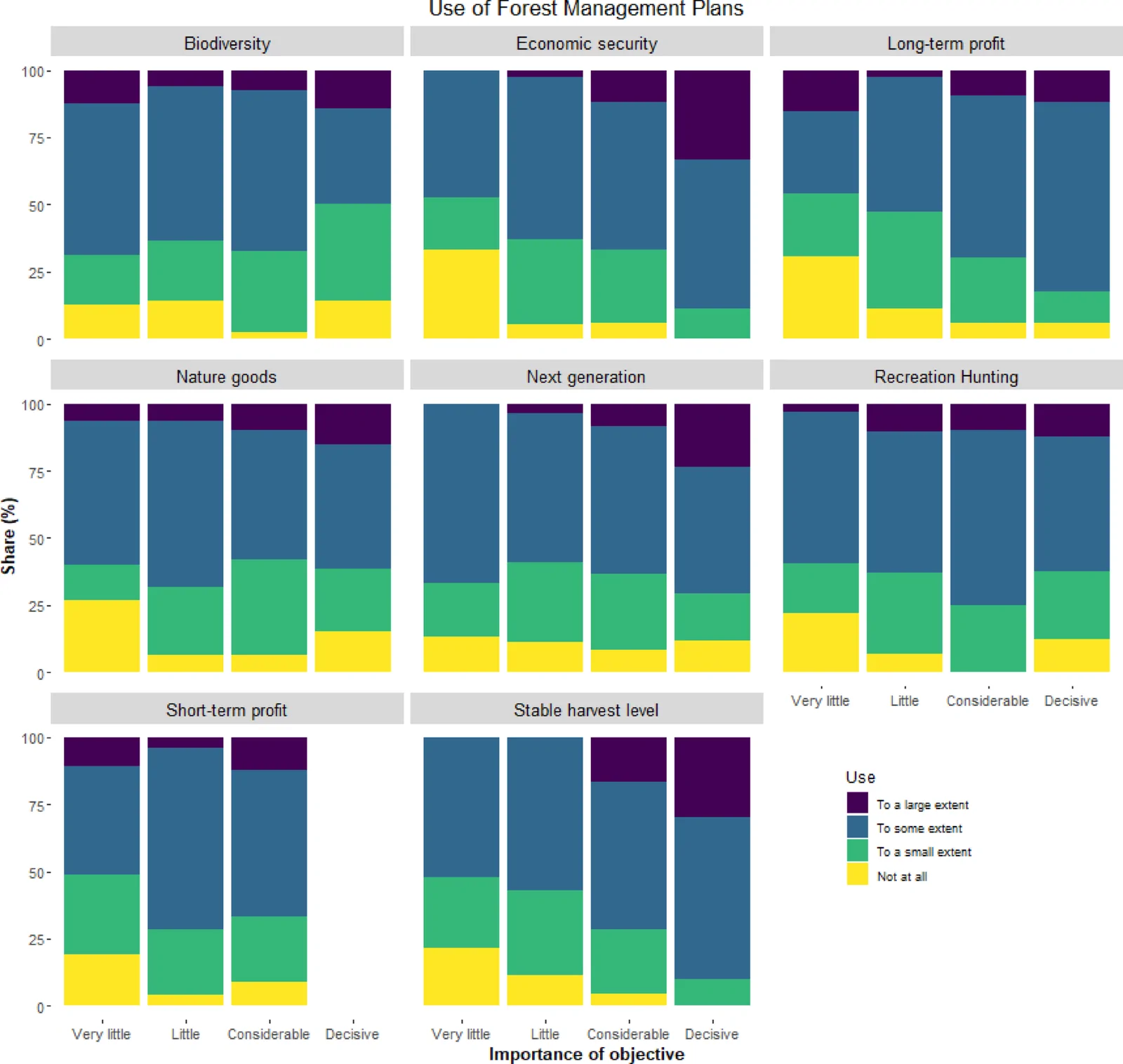
Use of FMP based on importance of ownership objectives.

More emphasis on economic security, next generation’s needs and stable harvest level implied more use of FMP (Fig. 4). We assessed whether the use of FMP varies with ownership objective using Spearman’s rank-order correlation. We found statistically significant relationships between economic security objective and the use of FMP to plan harvest volumes over several years (rho = 0.285; p-value < 0.01), between long-term profit as management objective and the use of FMP (rho = 0.239; p-value < 0.01) and between stable harvest level as management objective and the use of FMP (rho = 0.326; p-value < 0.01). The tests showed no statistically significant association between the use of FMP and biodiversity protection, nature goods, recreation/hunting, next generation needs and short-term profits as ownerships objectives.

We constructed an ordinal regression model to find factors that explained increasing use of FMP. Owners who reported economic management objectives used the FMP significantly more than others. For every unit increase in economic ownership objectives, the odds of using the FMP to determine harvest levels increased by 34% (Table 5). Biodiversity objectives did not influence the use of FMP. A change in productive forest area of 2.72 times implies 44% higher odds of more use of the FMP. Having received instruction about the use of FMP and assessing the FMP predictions to be correct, each increased the odds of using the FMP by 148% and 92%, respectively; however, the latter association was only marginally significant. Gender did not impact the usage of FMP for determining harvest levels, while older owners used the plan more than younger owners. Nagelkerke’s pseudo R^2^ equaled 0.30.

**Table 5:** Ordinal regression model of using FMP to determine harvest volumes. OR = odds ratios.

	Value	SE	Z value	*p* value	OR
Forest area (log)	0.366	0.193	1.899	0.058	1.442
FMP Instruction (Yes)	0.910	0.417	2.184	0.029	2.484
Economic objectives	0.295	0.089	3.306	0.001	1.343
Biodiversity objective	− 0.054	0.212	− 0.254	0.800	0.948
Assessment of predictions (Yes)	0.650	0.384	1.690	0.091	1.915
Gender (M)	0.744	0.462	1.612	0.107	2.105
Owner age	0.034	0.016	2.073	0.038	1.034
1|2	5.002	1.746	2.865	0.004	
2|3	7.077	1.801	3.930	0.000	
3|4	10.614	1.957	5.423	0.000	

Summing up, our results supported H1: Forest owners with economic objectives have higher harvest intensity than others. Our findings did not support H2: The use of FMP increases harvest intensity. We found support for H3: Forest owners with economic ownership objectives use FMP more than others.

## Discussion

By using a unique dataset to compare property-level actual harvest levels to predicted volumes over the same period, we could identify timber price, ownership objectives, forest owner and property characteristics that impact harvest intensity over a period of 5–7 years after the acquisition of FMP among forest owners who purchased this decision-support tool. However, the stated use of FMP itself did not impact harvest intensity. Forest area impacted also on the use of FMP, but in the opposite direction as for harvest intensity. The findings indicate that over the analyzed time span, owners with smaller properties harvest considerably more intensively compared to those with larger properties, based on the log of forest area. Nationally, harvest volumes equal 1.6 m^3^ per hectare and year, with no clear trends across property size^26^. However, the average site productivity is considerably better on smaller properties^42^ suggesting that over time, harvest levels would be negatively correlated with property size. There are few comparable analyses, but a previous study suggests that for all forest properties in Norway taken together, harvest levels sum up to 65% of the maximum sustainable yield on the properties above 500 hectares with the corresponding number being 40% on properties smaller than 100 hectares^43^. Together, the previous findings indicate an accumulation of forest mature to harvest on small properties. However, in our sample, harvest intensity is negatively correlated with productive forest area. With longer time series, this pattern may change. It should be noted that the median harvest intensity is 1.0, showing that harvest level below the predictions is as common as a harvest level above the predicted level.

Of the fifteen properties with harvest intensity exceeding 3, only one was larger than 140 hectares. In the national population of NIPF owners, 13% harvest timber within a year; among the smallest properties, only 2% harvest yearly^26,44^. We speculate that new FMPs trigger harvest to a higher degree on smaller than on larger properties with the relatively short time span in the analysis enhancing this effect. In our sample, the mean harvest intensity is 1.9, almost double the predictions’ level. Total harvest level summed over all properties divided by total predicted volume summed over all properties equaled 1.24, indicating an overall over-harvest compared to the maximum sustainable yield. Harvest levels have generally been below maximum sustainable yields in Norway in the last decades^45^. The findings could be explained by high timber prices in the analyzed period or growing concerns about retaining forests above maturity age due to increased rate of drought and calamities. Previous studies have found that owners with larger land are less price-sensitive^46^, which could be explained by their higher dependency on stable timber incomes than smaller owners who can afford to be more opportunistic. We expected to identity a regional effect, but it vanished with timber price, due to the structural, geographical differences in timber prices^30^.

We found that ownership objectives impact the harvest level over several years, with biodiversity protection objective having an effect of similar magnitude as having next generation’s needs as ownership objective, both of negative significance. The importance of ownership objectives for harvest decisions supports the findings from other revealed-preferences studies in larger and other geographies^12,47,48^. However, short-term profit objective did not impact harvest levels. The impacts of owner age align well with earlier studies, as older owners may have less debt^49^. We found that engagement is an important predictor for harvest intensity, which could be attributed to engaged owners being more aware of high timber prices, mature forest and available volumes.

Previous studies that have investigated the impacts of FMPs on harvest have included FMP as a binary variable, finding it leading to more harvest^13,50^. While having a FMP may indicate engagement and knowledge about the property and forestry, our sample only included owners with FMP. In our sample, the stated use of plans did not impact harvest intensity either positively nor negatively. 82% of the sampled owners used FMP to decide the harvest level to a small or to some extent. Thus, FMP serve as a guidance to the owners, while they choose harvest levels to maximize utility by adapting to timber prices. Most FMP in Norway propose harvest and management schematically based on timber-production with no adaptation to the individual owner’s preferences, while we found that forest owners with objectives such as long-term profit, stable harvest levels and economic security use the FMP more than others. Thus, current FMPs seem to be particularly well suited to this group of owners. However, for the population of NIPF owners, conservation objectives are almost as important for forest owners as economic goals, and social and psychological objectives are more important than economic objectives^12^. Owners use FMP more if they deem that it is adapted to their management objectives^16^. Consequently, the gap between the current FMPs and ownership objectives may limit the use of FMPs among certain groups of owners. The importance of adapting FMP to owner’s need may grow as owners value amenities increasingly more and the forest owner population is becoming more diverse^51,52^.

FMP data collection and design are traditionally in about 15 years’ cycles with methods and scope regulated by public authorities. In the future, FMP for even-aged monocultures may to a higher extent be based on remotely sensed data with lower costs and more flexibility and opportunities for individual design which in turn could enhance use rate of FMPs among multi-objective owners. It is also worth noting that the harvest predictions are based on empirical, deterministic models of growth and mortality. In Norway, tree mortality is increasing^53^, and high mortality or reduced vitality may have lead forest owners to harvest more timber in this period. Preventive harvest to avoid mortality is hard to detect, but future studies would benefit from attempting to investigate this matter, as this is a likely increasing factor in harvest decisions due to global change.

The readers should be aware of the study’s limitations. The sample is relatively small, and the distribution of harvest intensity is left-tailed. However, we ran the harvest intensity model with censored data to harvest intensity of 10 and 5, respectively, which yielded similar results as the presented model. The next generation objective was no longer significant in the model based on this dataset, while the other variables maintained their sign of coefficient and significance level. The R^2^ value of 0.13 for the harvest intensity model indicates that there is considerable variation in the dataset not being captured by the model. While it is at a similar level as another study using forest inventory data to explain harvest levels^15^, it is lower than studies using only empirical timber harvest data but no timber volume data^12,13^. With the nascent literature using richer forest inventory data to explain forest owner behavior, it is noteworthy that these two models exhibit lower explanatory power than models that only consider forest area. This could possibly be due to the relatively short time span in our analysis and that forest owners see inventory data as a long-term ceiling rather than a short-term recommendation of harvest level or that demand-side and biological factors not captured by the harvest predictions are the main driving factors in steering harvest levels. However, with a minimum of studies using forest inventory data to explain harvest behavior, these hypotheses and others warrant to be tested in future studies.

Other studies have discussed the two-way relationship between the purchase of FMP and harvesting^16^. Our sample only selected owners who already have purchased FMP, and we have examined whether using the FMP leads to more harvesting. However, positive feedback, i.e., that harvesting triggers forest owners to use the already purchased FMP more, cannot be ruled out. In-depth surveys or interviews could reveal more of the time dynamics of FMP purchase and use versus timber harvesting.

The mean productive forest area in our data is 193.5 ha, considerably larger than the mean forest area for Innlandet NIPF owners of 60 hectares^31^ and the corresponding national number of 46 ha^26^. All owners in the sample have FMP in contrast to the national level where 37% of NIPF owners have FMP^16^. On the national level, the mean forest area of owners with FMP is 92 ha, thus, our sample is largely constituted of semi-professional forest owners. While the average age of the respondents of 60 years is close to the number for the national population, 20% females among the respondents is lower than the national share of 25%^16^. We believe the rather technical questions related to FMP deterred participation from many small forest owners. The FMP predictions have a dual role in the study of forest management. First, they serve as a recommendation of a ‘reasonable’ harvest level toward the forest owners. Second, they are used to calibrate actual harvest level against a potential, given the constraint of non-declining harvest level. The analyzed time span of 5–7 years is short. Following up these owners in some years would give more nuanced insight into how harvest levels deviate from harvest potentials and how management objectives shape management.

## Conclusion

We have found that among semi-professional NIPF owners in Innlandet, Norway, harvest intensity calculated as the ratio of actual harvest volumes to predicted volumes in the same period, decreases with property size and preferences for biodiversity conservation and the next generation’s needs. The use of FMP did not influence harvest intensity; however, forest owners’ use of the FMP was affected by ownership objectives and forest area. NIPF owners’ management is shaped by their preferences and objectives, knowledge, markets and the biophysical attributes of their forest. A better understanding of these interactions is important for policymaking and extension services.

NIPF owners manage vast areas of forestland and their harvest and management decisions shape these ecosystems and the supply of timber and other ecosystem services. However, there is a paucity of studies that investigate willingness to harvest by analyzing actual harvest. The study’s main novelty lies in comparing actual harvest levels with predicted volumes for the same time period, thus considering each property’s biophysical resource using data based on stand-level inventories. Although the data sample is small and represents only a fraction of NIPF owners, we have demonstrated a new combination of biological, social and economic data. We believe our approach demonstrates a useful method for revealed-preferences studies that account for the forest resource base relevant to the decision-maker. Such approaches can be valuable for examining actual forest owner behavior.

## Supplementary Information

Below is the link to the electronic supplementary material.


Supplementary Material 1


## Data Availability

The data that support the findings of this study are partly available from the University of Inland Norway (INN), but restrictions apply to their availability due to protect privacy (cf. the European Union’s General Data Protection Regulation (GDPR)).
