# Supplementary material for: Timber harvesting intensity and use of decision-support tools among semi-professional private forest owners: a Norwegian case study
Source: Sci Rep. 2026 Jun 15;16:27316. doi: 10.1038/s41598-026-55795-0 (PMC13530187; doi:10.1038/s41598-026-55795-0)
Supplement: Supplementary file 1 [file 41598_2026_55795_MOESM1_ESM.pdf]

Supplementary S1: Forest owner questionnaire in Norwegian

Original questionnaire in Norwegian used in to survey forest owners in the research article "*Property-level harvest intensity and use of decision-support tools*".

Betydning av skogbruksplan i beslutninger om hogst

Her følger spørreundersøkelsen for skogeiere i XXX kommune. Under følger informasjon om personvern og håndtering av data. Samtykke kan gis på neste side, og deretter begynner undersøkelsen.

Personvern og håndtering av data

Dataene vil brukes i et forskningsprosjekt, basert på disse tre datakildene:

1) Denne spørreundersøkelsen

2) Forslag til hogstvolum fra skogbruksplanen Allma, fra Planavdelingen i Glommen Mjøsen Skog

3) Data om avvirket tømmervolum fra Statsforvalteren i Innlandet

Når du svarer på undersøkelsen, blir du bedt om å oppgi navn, fødselsdato og adresse og samtykke i at vi kobler data om deg som eier og din eiendom mellom disse tre datakildene. Det er for at vi skal kunne koble ditt svar til din eiendom.

Deltagelse er frivilling. Hvis du ikke ønsker å delta, svarer du ikke på undersøkelsen. Du kan når som helst trekke deg og du kan uten begrunnelse kreve at dine data slettes, også etter at du har svart på undersøkelsen.

Vi har fått godkjenning fra Statsforvalteren i Innlandet til bruk av hogstdata dersom du samtykker til det, og fra Sikt (tidligere Norsk senter for forskningsdata) for å gjennomføre undersøkelsen slik vi beskriver her. Identifisering av eier gjøres kun for å koble de tre datakildene. Etter dette vil identifiserende informasjon ligge separat fra de andre dataene, i en egen passordbeskyttet mappe på Høgskolen i Innlandets server, hvor kun forskerne i prosjektet har tilgang. Denne slettes 1. juni 2025. Forskerne har taushetsplikt og vil aldri videreformidle opplysninger om enkeltpersoner eller eiendommer til andre, og følger Høgskolens retningslinjer for behandling av personopplysninger i forskning (https://www.inn.no/bibliotek/forskningsstotte/forskningsdata/retningslinjer-for-forskningsdata/index.html).

Resultatene vil bare offentliggjøres på et sammenstilt nivå som artikler og presentasjoner hvor det ikke er mulig å identifisere verken enkelte eller små grupper av eiendommer eller eiere. Ta kontakt hvis du ønsker tilsendt slike artikler.

Forskningen ledes av Høgskolen i Innlandet, og utføres i samarbeid med Glommen Mjøsen Skog SA. Det er to forskere i prosjektet, Hanne K. Sjølie (ansvarlig for undersøkelsen) og Ane C. Tange. Har du spørsmål om undersøkelsen, hvordan dine data blir behandlet eller ønsker å trekke deg, kan disse kontaktes på epost hanne.sjolie@inn.no, tlf. XXXXXX og ane.tange@inn.no, tlf. XXXXXX.

Aktivt samtykke

Vi trenger ditt samtykke til punkt 1-3 under for å bruke data for din eiendom i undersøkelsen

1) Hogstdata fra Skogfondsregisteret for perioden 2007-2023

2) Forslag til hogstvolum fra skogbruksplanen fra Glommen Mjøsen Skog

3) Data mellom dette spørreskjemaet og de to ovennevnte datakildene kobles

Samtykker du til at vi innhenter data som beskrevet i punkt 1-3 over?

Sett ett kryss

Ja, jeg samtykker

Nei, jeg samtykker ikke

Takk for at du samtykker. Trykk Neste side for å bli sendt til spørreskjemaet.

Hvis du allikevel ønsker å svare på undersøkelsen, kan du endre svar på samtykket. Hvis ikke, kan du lukke dette vinduet. Takk for at du tok deg tid.

Først trenger vi noen personopplysninger om deg. Som beskrevet, ville disse kun brukes for å koble dine svar til din eiendom.

Navn

Hva er din folkeregistrerte adresse?

Hva er ditt kjønn?

Sett ett kryss

Mann

Kvinne

Annet/ønsker ikke å svare

Hva er ditt fødselsår?

Ta utgangspunkt i den eiendommen du kjøpte skogbruksplan for når du svarer på resten av spørreskjemaet.

Hvor aktiv er du i den løpende forvaltningen av din skog?

Sett ett kryss per linje

Jeg følger med på skogens tilstand

I ingen grad

I liten grad

I noe grad

I stor grad

Jeg følger med på tømmerpriser

I ingen grad

I liten grad

I noe grad

I stor grad

Jeg følger med på faglige spørsmål om skogbruk

I ingen grad

I liten grad

I noe grad

I stor grad

Jeg er opptatt av riktig skjøtsel av skogen

I ingen grad

I liten grad

I noe grad

I stor grad

Beslutninger om hogst

Nå følger noen spørsmål om årsaker til hogstvolum etter at du kjøpte skogbruksplanen. Årsakene kan være knyttet til dine ønsker og til forslag til hogstvolum. Med forslag til hogstvolum mener vi anbefalinger om årlig avvirkningsvolum som er oppgitt i skogbruksplanen.

Bruk av skogbruksplanen og forslag til hogstvolum

Hvordan bruker du skogbruksplanen til å planlegge hogst og skogskjøtselstiltak?

Sett ett kryss per linje

Planlegge hogst for flere år

I ingen grad

I liten grad

I noe grad

I stor grad

Når det leveres skogbruksplaner til en kommune, er det ofte en del ekstra aktivitet knyttet til lansering. Deltok du i noen av disse aktivitetene ved eller i etterkant av kjøp av skogbruksplanen?

Sett ett eller flere kryss

Skogdag

Opplæring i bruk av skogbruksplanen fra leverandør eller skogbruksleder/virkeskjøper

Opplæring i bruk av skogbruksplanen fra Skogkurs

Informasjon fra skogbrukssjef

Ekstra informasjon om lønnsomhet ved tiltak i skogbruket

Ingen av disse

Annet, spesifiser under

Dine målsetninger for skogen

Under følger mulige målsetninger i forvaltningen av skog. Hvor stor betydning har disse målene for deg i forvaltningen av din skog?

Sett ett kryss per linje

Kortsiktig tømmeravvirkning og økonomisk overskudd

Svært lite betydning

Noe betydning

Ganske stor betydning

Avgjørende betydning

Langsiktig tømmeravvirkning og økonomisk overskudd

Svært lite betydning

Noe betydning

Ganske stor betydning

Avgjørende betydning

Økonomisk sparing eller sikkerhet

Svært lite betydning

Noe betydning

Ganske stor betydning

Avgjørende betydning

Jevn avvirkning

Svært lite betydning

Noe betydning

Ganske stor betydning

Avgjørende betydning

Neste generasjons behov

Svært lite betydning

Noe betydning

Ganske stor betydning

Avgjørende betydning

Hensyn til jakt, fiske og friluftsliv

Svært lite betydning

Noe betydning

Ganske stor betydning

Avgjørende betydning

Biologisk mangfold

Svært lite betydning

Noe betydning

Ganske stor betydning

Avgjørende betydning

Et bredt spekter av naturgoder

Svært lite betydning

Noe betydning

Ganske stor betydning

Avgjørende betydning

I hvilken grad mener du forslagene til hogstvolum gir et riktig bilde av et fornuftig avvirkningsnivå?

Sett ett kryss

Forslagene gir et altfor høyt avvirkningsnivå (over 30 prosent for høyt)

Forslagene gir et for høyt avvirkningsnivå (mellom 10 og 30 prosent for høyt)

Omtrent riktig nivå (mellom 10 prosent for høyt og 10 prosent for lavt)

Forslagene gir et for lavt avvirkningsnivå (mellom 10 og 30 prosent for lavt)

Forslagene gir et altfor lavt avvirkningsnivå (over 30 prosent for lavt)

Vet ikke

I hvor stor grad bruker du forslag til hogstvolum til å styre avvirkningsnivået over flere år?

Sett ett kryss

I ingen grad

I liten grad

I noe grad

I stor grad

Supplementary S2: Translated questionnaire in English

The generative AI- service from SIKT[1] has been used to translate the questionnaire used in the research article "*Property-level harvest intensity and use of decision-support tools*" into English. Deviation from the terminology used in the article may occur.

The Role of Forest Management Plans in Harvest Decisions

Below is the survey for forest owners in XXX municipality. The following section provides information about data privacy and data handling. Consent can be provided on the next page, after which the survey will begin.

Data Privacy and Data Handling

The data will be used in a research project based on the following three data sources:

1) This survey

2) Proposed harvest volumes from the forest management plan Allma, provided by the Planning Department at Glommen Mjøsen Skog

3) Data on harvested timber volumes from the County Governor of Innlandet

When responding to the survey, you will be asked to provide your name, date of birth, and address, and to consent to us linking data about you as an owner and your property across these three data sources. This is necessary to connect your response to your property.

Participation is voluntary. If you do not wish to participate, simply do not respond to the survey. You may withdraw at any time, and you can, without providing a reason, request that your data be deleted, even after you have completed the survey.

We have received approval from the County Governor of Innlandet for the use of harvest data, provided you consent to it, and from Sikt (formerly the Norwegian Centre for Research Data) to conduct the survey as described here. Identification of the owner is done solely to link the three data sources. After this, identifying information will be stored separately from the other data, in a password-protected folder on the server at Inland Norway University of Applied Sciences, accessible only to the researchers involved in the project. This information will be deleted on June 1, 2025.

The researchers are bound by confidentiality and will never share information about individuals or properties with others. They also adhere to the university's guidelines for processing personal data in research (https://www.inn.no/english/library/research-support/research-data/guidelines-for-research-data/index.html).

The results will only be published at an aggregated level, such as in articles and presentations, where it will not be possible to identify individual properties, owners, or small groups of properties or owners. Please contact us if you would like to receive copies of such articles.

The research is led by Inland Norway University of Applied Sciences and is conducted in collaboration with Glommen Mjøsen Skog SA. There are two researchers involved in the project: Hanne K. Sjølie (responsible for the survey) and Ane C. Tange. If you have questions about the survey, how your data will be handled, or if you wish to withdraw, you can contact them at the following: - Email: hanne.sjolie@inn.no, Phone: XXXXXX and Email: ane.tange@inn.no, Phone: XXXXXX

Active Consent

We need your consent for points 1-3 below in order to use data related to your property in this survey:

1) Harvest data from the Forest Fund Register for the period 2007-2023

2) Proposed harvest volumes from the forest management plan provided by Glommen Mjøsen Skog

3) Linking data between this survey and the two data sources mentioned above

Do you consent to us collecting data as described in points 1-3 above?

Please check one box:

☐ Yes, I consent

☐ No, I do not consent

Thank you for providing your consent. Click "Next Page" to proceed to the survey.

If you still wish to respond to the survey, you can change your consent response. Otherwise, you may close this window. Thank you for taking the time.

First, we need some personal information about you. As described, this information will only be used to link your responses to your property.

Name

What is your registered address?

What is your gender?

Please check one box:

☐ Male

☐ Female

☐ Other/Prefer not to answer

What is your year of birth?

Base your answers for the rest of the questionnaire on the property for which you purchased a forest management plan.

How actively involved are you in the ongoing management of your forest?

Please check one box per line:

I watch the state of the forest

☐ Not at all

☐ To a small extent

☐ To some extent

☐ To a large extent

I observe timber prices

☐ Not at all

☐ To a small extent

☐ To some extent

☐ To a large extent

I study technical questions about forestry

☐ Not at all

☐ To a small extent

☐ To some extent

☐ To a large extent

I care about proper management of the forest

☐ Not at all

☐ To a small extent

☐ To some extent

☐ To a large extent

Decisions about Harvesting

The following are some questions about the reasons for harvest volumes after you purchased the forest management plan. The reasons may be related to your preferences and the proposed harvest volumes. By "proposed harvest volumes," we mean the recommendations for annual harvest volumes specified in the forest management plan.

Use of the Forest Management Plan and Proposed Harvest Volumes

How do you use the forest management plan to plan harvest and forest management measures?

Check one box per line

Plan harvesting for multiple years

☐ Not at all

☐ To a small extent

☐ To some extent

☐ To a large extent

When forest management plans are delivered to a municipality, there is often additional activity associated with the launch. Did you participate in any of these activities at or after purchasing the forest management plan?

Check one or more boxes

☐ Forest day

☐ Training in the use of the forest management plan from the supplier or forestry manager/timber buyer

☐ Training in the use of the forest management plan from Skogkurs

☐ Information from the forestry officer

☐ Additional information about the profitability of forestry measures

☐ None of these

☐ Other, specify below

Your Objectives for the Forest

Below are potential objectives in forest management. How important are these objectives to you in managing your forest?

Check one box per line

Short-term timber harvesting and economic profit

☐ Very little importance

☐ Some importance

☐ Considerable importance

☐ Decisive importance

Long-term timber harvesting and economic profit

☐ Very little importance

☐ Some importance

☐ Considerable importance

☐ Decisive importance

Economic savings or security

☐ Very little importance

☐ Some importance

☐ Considerable importance

☐ Decisive importance

Stable harvesting levels

☐ Very little importance

☐ Some importance

☐ Considerable importance

☐ Decisive importance

Next generation's needs

☐ Very little importance

☐ Some importance

☐ Considerable importance

☐ Decisive importance

Consideration for hunting, fishing, and outdoor recreation

☐ Very little importance

☐ Some importance

☐ Considerable importance

☐ Decisive importance

Biodiversity

☐ Very little importance

☐ Some importance

☐ Considerable importance

☐ Decisive importance

A wide range of nature goods

☐ Very little importance

☐ Some importance

☐ Considerable importance

☐ Decisive importance

To what extent do you believe the proposed harvest volumes provide an accurate picture of a reasonable harvesting level?

Check one box

☐ The proposals suggest a harvesting level that is far too high (over 30 percent too high)

☐ The proposals suggest a harvesting level that is too high (between 10 and 30 percent too high)

☐ Approximately the right level (between 10 percent too high and 10 percent too low)

☐ The proposals suggest a harvesting level that is too low (between 10 and 30 percent too low)

☐ The proposals suggest a harvesting level that is far too low (over 30 percent too low)

☐ Don't know

To what extent do you use the proposed harvest volumes to guide the harvesting level over multiple years?

Check one box

☐ Not at all

☐ To a small extent

☐ To some extent

☐ To a large extent
